# A Journey Through the Landscapes of Small Particles in Binary Colloidal Assemblies: Unveiling Structural Transitions from Isolated Particles to Clusters upon Variation in Composition

**DOI:** 10.3390/nano9070921

**Published:** 2019-06-26

**Authors:** Valeria Lotito, Tomaso Zambelli

**Affiliations:** Laboratory of Biosensors and Bioelectronics, Institute for Biomedical Engineering, ETH Zurich, Gloriastrasse 35, 8092 Zurich, Switzerland

**Keywords:** binary colloids, colloidal monolayers, amorphous colloidal assemblies, air/water interface self-assembly, disordered colloidal assemblies, sparse colloidal assemblies, pattern analysis

## Abstract

Two-dimensional (2D) amorphous binary colloidal assemblies composed of particles of two different sizes are characterized by the loss of hexagonal close-packing for larger particles, occurring when the size ratio between small (S) and large (L) particles $d_{S}/d_{L}$ exceeds a certain threshold value. For moderately low particle number ratios $N_{S}/N_{L}$ large particles still retain a denser arrangement with transitions from hexagonal symmetry to the coexistence of different types of symmetries as $N_{S}/N_{L}$ progressively departs from 0 to higher values. On the other hand, small particles reveal sparser arrangements: shape identification and quantification of structural transitions in small particle arrangements appear particularly challenging. In this article, we investigate their shapes and transitions for amorphous binary colloidal particles assembled at the air/water interface. For the quantitative characterization of the evolution in particle arrangements for $N_{S}/N_{L}$ variable between 0.5 and 2, we develop an innovative procedure for morphological analysis, combining Minkowski functionals, Voronoi diagrams and ad hoc techniques to recognize and classify specific features. Such a powerful approach has revealed a wide variety of landscapes featuring isolated particles, dimers, chains, small clusters evolving with the colloidal suspension composition. Our method can be applied to the analysis of spatial configurations of sparse colloidal patterns obtained in different conditions.

## 1. Introduction

Quasi-spherical colloidal particles of two different sizes can give rise to two-dimensional (2D) binary assemblies of different types according to their composition [1]. In particular, they can result in periodic assemblies in which large (L) particles preserve a hexagonal arrangement and small (S) particles fill the interstices between L particles or are placed along the line connecting two neighboring L particles in a periodic fashion [2,3,4]. Other types of regular arrangements, for instancewith periodic square or rhombic symmetry, are, in principle, possible [5], but in real experiments they are typically encountered over tiny areas [6,7]. More often, if the more ordered and regular hexagonal symmetry is not observed, more amorphous and disordered patterns can be generated, in which L particles lose the hexagonal symmetry and show a coexistence of different symmetries, e.g., hexagonal, square and rhombic phases, geometries of transition between the different symmetries, random tiling [5,8,9,10,11,12]. In some cases, segregation of S and L particles can even occur in which S and L particles separately assemble in areas with hexagonal symmetry [2,5,13].

The transition between different periodic configurations within the regime of preservation of hexagonal order for L particles as well as the transition between the regime of preservation and the regime of the loss of hexagonal order depend on the ratio between particle diameters (particle size ratio $d_{S}/d_{L}$) and on the ratio between the number of particles (particle number ratio $N_{S}/N_{L}$); the conditions for the transition between periodic and aperiodic patterns have been studied theoretically and experimentally [2,3,4,5,12,14].

The morphology of binary colloidal assemblies in the regime of preservation of hexagonal symmetry has been extensively investigated to individuate the different periodic arrangements of S particles for variable $N_{S}/N_{L}$, using different analysis tools [4,15]. Periodic binary assemblies have found very diverse applications from the engineering of phononic materials with tunable sound wave propagation [16,17], to templated fabrication of plasmonic structures [18].

More recently, also amorphous binary colloidal assemblies have attracted the attention of scientists. The introduction of disorder has a dramatic impact on material properties and important implications, for instance, in the interactions of a colloidal material with light or sound [11,17,19,20,21,22,23]. Restricting the attention to optical properties, it has been observed that ordered systems interact with light via collective modes resulting in diffraction, while completely disordered systems are dominated by Mie resonances of individual particles and random scattering; in the intermediate regime, collective modes, Mie resonances and near field interactions between individual scatterers all play a role; the transition between the different optical behaviors has been observed in binary colloidal assemblies for variable particle composition [11].

Despite the potential interest of binary colloidal assemblies in the regime of loss of hexagonal order in practical applications, the analysis of their morphology poses more challenges in comparison to ordered assemblies. In fact, traditional analysis tools (such as the pair correlation function), which prove to be successful for the analysis of ordered assemblies [4,24,25,26], provide little clue to a comprehensive and thorough description of the colloidal morphology and fail to deliver a quantitative characterization of the patterns generated in these more disordered assemblies [12,21]. For such more complex structures, more sophisticated analysis tools need to be devised. To this end, a combined approach merging computational geometry and computational topology has been developed, able to supply a quantitative description of the evolution of patterns generated by L particles in amorphous binary assemblies obtained at the air/water interface at a value of $d_{S}/d_{L}$ slightly above the theoretical threshold for the occurrence of a periodic square packing and for increasing $N_{S}/N_{L}$ [12]. A key element in such a quantitative classification has been represented by the use of persistent homology, a tool applied very recently to the analysis of granular and colloidal assemblies [27,28,29,30]. In particular, the use of persistence diagrams, which allows the study of the characteristics of the “holes” constructed starting from a set of points (a point cloud formed, in the case of colloidal particles, by the centroids of the particles), has revealed the relative weight of hexagonal, square and rhombic arrangements of L particles controllable via the composition of the colloidal suspension [12].

For moderately low particle number ratios, L particles still retain a denser grouping, even if with a packing fraction that progressively declines and with a variety of geometrical arrangements that is continuously enriched as the concentration of S particles increases. On the other hand, S particles are more sparsely disposed; a visual inspection suggests the presence of different patterns going from isolated S particles especially for lower $N_{S}/N_{L}$ to small chains, rings and clusters of S particles in the interstices between L particles becoming more frequent at higher S particle concentrations. Fingerprints of such structural transitions have been found by applying the analysis of persistence diagrams to the sole S particles [12]. Specific arrangements of S particles (e.g., consisting of a single S particle surrounded by four L particles with centroids located at the vertices of a square) have been individuated by resorting to the study of bond orientational order parameters and bond length deviations [8]. However, all the different spatial arrangements of S particles have not been fully and quantitatively classified and characterized.

Finding an appropriate tool for the analysis of the spatial configurations of S particles is of interest not only for binary colloids. Similar structures consisting of chains or rings of colloidal particles can be found in different contexts within the field of colloid science. Chaining has been observed in self-assembly based on the use of electric and magnetic fields [31,32,33]. More recently, ring-like assemblies have been examined within the framework of block copolymer-mediated self-assembly of nanoparticles [34]. Anisotropic assembly phases, in particular chains, have been found in the investigation of the interfacial behavior of colloidal particles in a Langmuir trough in the presence of surfactants, block copolymers and proteins [35,36]; chaining has been witnessed under specific experimental conditions in terms of the spreading agent, subphase pH and conditions of injection of a colloidal suspension to the air/water interface [37]. In general, the search for more complex colloidal arrangements different from the more common hexagonal pattern is an active research area subject to lingering efforts of characterization and control, as it could pave the way for engineering materials with more exotic properties and open up new horizons for surface patterning. For instance, chaining has been shown to influence the haze factor of a particle layer [36]. Hence, the development of tools for the recognition and quantitative analysis of such shapes is of primary importance for the characterization of such novel types of assemblies.

For sparse structures exhibiting self-similarity, such as fractal-like aggregates, the computation of fractal dimension has been used in previous studies [31,38,39], but such an analysis does not provide a classification and quantification of the different structures and spatial arrangements and requires care in the choice of the computation method and parameters [40]. Concerning cluster recognition, recently, a method has been developed to distinguish between areas with low-density and high-density packing [41]. However, such an approach is not suitable for the identification of chains, rings and clusters made up of few particles.

In this article, we develop and describe a novel method for the quantitative analysis of such structures and we apply it to the analysis of S particles in binary colloids assembled at the air/water interface. More specifically, this approach allows the accomplishment of a three-fold investigation:

- Examination of the configurations assumed by S particles, i.e., identification of isolated particles, dimers, chains, rings and clusters;
- Examination of the configurations assumed by L particles relative to S particles in order to identify specific recurrent patterns characterized by angular and radial uniformity of L particles with respect to S particles (for instance, isolated S particles in the interstices between three L particles arranged in an equilateral triangle and between four L particles arranged in a square or S particle dimers in the interstices between four L particles arranged in a rhombus);
- Examination of the configurations assumed by S particles relative to L particles, e.g., determination of L particles “caged” by S particles and analysis of the angular and radial uniformity of S particles around L particles.

As will be shown, the technique combines neighborhood analysis, Voronoi tessellation, shape factor calculation and Minkowski functionals. This method allows a quantification of the diverse patterns encountered in such assemblies and the analysis of the structural evolution of such complex colloidal landscapes for variable S particle concentration. The method is inherently a powerful approach suitable for the analysis of variegated sparse colloidal patterns obtained under different conditions.

## 2. Materials and Methods

### 2.1. Colloidal Self-Assembly

Binary colloidal suspensions were obtained starting from polystyrene (PS) particles purchased from Thermo Scientific (Waltham, MA, USA) (5000 Series Polymer particles packaged as aqueous suspensions at 10 wt % solids) of nominal diameters equal to $d_{S}=220\;\mathrm{nm}$ and $d_{L}=500\;\mathrm{nm}$ and a coefficient of variation (CV) of less than 3%. For the assembly of binary colloids, we used interfacial self-assembly, which has emerged as an effective technique to assemble diverse types of objects, from particles of different materials and shapes to semiconductor nanocrystals and exfoliated graphene films [1,4,24,42,43,44,45,46,47,48]. In particular, colloidal assemblies were prepared at air/water interface by the technique of surface confinement and water discharge described in [4,24]. Colloidal suspensions with variable $N_{S}/N_{L}$ values were mixed 1:1 with ethanol. A tilted glass slide partially immersed in a glass container filled with water and a small amount of surfactant (0.1 mM sodium dodecyl sulphate) was used to supply colloidal particles to the air/water interface within a surface area confined by a nitrile butadiene rubber (NBR) ring kept in a fixed position by three polytetrafluoroetylene cylindrical rods inserted in a circular base made of the same material and placed at the bottom of the glass container. Prior to particle injection, a sample was placed beneath the water surface within the area confined by the NBR ring on a sample holder inserted in the circular base. The glass container was equipped with a tap. After self-assembly at the air/water interface, a colloidal monolayer was smoothly transferred onto the target substrate by opening the tap and letting water flow out of the container. Binary assemblies with $N_{S}/N_{L}$ values equal to 0.5, 1, 1.5 and 2 were produced. The particle number ratio was calculated as described in [12].

### 2.2. Characterization and Analysis of Particle Configurations

Zeiss Ultra 55 and Zeiss Ultra Plus scanning electron microscopes (Oberkochen, Germany) were used to acquire scanning electron microscopy (SEM) images of the colloidal assemblies after transfer on the samples and deposition of a thin conductive layer by electron beam evaporation.

The coordinates of the centroids of L and S particles were determined by circle detection using algorithms based on the circular Hough transform and were used for the subsequent analyses.

For the first type of analysis, i.e., the examination of the configurations of S particle arrangements, only the coordinates of S particles were considered.

Voronoi diagrams were constructed using such coordinates as seeds for the tessellation, consisting in partitioning the space into cells $V_{j}$ associated to each seed (particle) j, such that the points associated to the seed j are closer to j than to any other seed (particle) in the space. The probability distribution of the area of such cells was investigated as well as the inter-particle distance $d_{\mathrm{SNN}}$ between the nearest neighbors determined with the Voronoi tessellation and its dual Delaunay triangulation derived by connecting by a straight edge two sites, whose corresponding Voronoi cells share an edge.

Subsequently, the different configurations were identified and classified based on the individuation of connected components in a binary image and on the definition of the neighborhood of each particle according to a minimum threshold distance R.

For the identification of connected components, a binary image is constructed starting from the centroids of the S particles, by placing a cover disc with a radius R/2 at each couple of coordinates of the particle centroids. Each connected component is made of one or more particles.

For the definition of neighborhood, we compute, for each particle j, the set of neighbors $N_{j}$ individuated, within this context, as all the particles that are within a distance R from the centroid of the particle j. The neighborhood cardinality, $\mathrm{card}\left(N_{j}\right)$, of the set $N_{j}$ defines the number of nearest neighbors of particle j according to the aforementioned definition of neighbors.

The different categories are classified according to the identification of connected components and on the definition of neighborhood. In particular, each of the connected components in the binary image constructed from the centroids of the S particles belongs to one the following categories that specify a configuration assumed by S particles:

- Isolated particles: a particle j belongs to this group if $\mathrm{card}\left(N_{j}\right)=0$, i.e., if it does not have any neighbor within a distance R from its centroid;
- Dimers: two particles j and k belong to this group if j has only the neighbor k within a distance R from its centroid and if k has only the neighbor j within a distance R from its centroid;
- Chains: $N_{\mathrm{part}}$ particles belong to a chain if they are such that each of the particles belonging to the chain has only two neighbors within a distance R from its centroid (neighborhood cardinality 2) except for two particles that have just one neighbor (neighborhood cardinality 1) and such a neighbor has a neighborhood cardinality equal to 2; roughly speaking, in a chain, all the particles have two neighbors except for the particles at the two extremes of the chain that have just one neighbor;
- Clusters: $N_{\mathrm{part}}$ particles belong to a cluster if they belong to a connected component that does not fall into any of the previous categories; within this set, we can distinguish loops; $N_{\mathrm{part}}$ particles belong to a loop if they all have a neighborhood cardinality equal to 2, i.e., if they all have two neighbors within a distance R from their centroid; a loop can be interpreted as a sort of “closed” chain or ring.

The different configurations were characterized in terms of the percentage of S particles belonging to each of the aforementioned categories and in terms of the size of chains, size of clusters classified as loops, and size of clusters other than loops (number $N_{\mathrm{part}}$ of particles belonging to each of these components). In addition, Minkowski functional analysis was carried out to scrutinize, in particular, the shape of clusters of a larger size including a number of particles $N_{\mathrm{part}}\geq 19$.

For the second and third analysis, i.e., the identification of the configurations of L particles relative to S particles and vice versa, the centroids of both S and L particles are considered.

The main goal of the second analysis is to determine the S and L particles involved in specific configurations, which have the potential for a periodic 2D space-filling, i.e., an isolated S particle between three L particles placed at the vertices of an equilateral triangle or between four L particles located at the vertices of a square or two S particles belonging to a dimer placed between four L particles in a rhombic arrangement.

It is noteworthy to observe that a square or an equilateral triangle arrangement of L particles is not sufficient for the individuation of such configurations. The crucial element is the position of the isolated S particle or S particle dimer within this equilateral triangle or square: the L particle centroids at the vertices of the equilateral triangle or of the square should satisfy specific conditions in terms of angular and radial uniformity with respect to the isolated S particle or to the S particle dimer. To this aim, first, a Voronoi tessellation is carried out to identify the number $N_{\mathrm{nnjLS}}$ of L nearest neighbors to isolated S particles and to the middle point of S particle dimers. Then, an approach based on the analysis of angular and radial uniformity of L particles around the isolated S particles or the S particle dimers is employed to find and characterize the particles involved in the previous configurations.

In particular, for the analysis of the angular uniformity, the bond orientational order parameter $\psi_{\mathrm{NLS}}^{\left(j\right)}$ relative to an isolated S particle or to the middle point of an S particle dimer j is computed as follows:

(1) $$\psi_{\mathrm{NLS}}^{\left(j\right)}=\frac{1}{N_{\mathrm{nnjLS}}}\sum_{k=1}^{N_{\mathrm{nnjLS}}}e^{\mathrm{Ni}\phi_{\mathrm{jk}}},$$

where $\phi_{\mathrm{jk}}$ is the angle of the line between the center of the isolated S particle (or the middle point of an S particle dimer) j and the $k^{\mathrm{th}}$ of its $N_{\mathrm{nnjLS}}$ L nearest neighbors with respect to an arbitrary fixed reference axis and N is an integer chosen according to the specific symmetry under investigation (for example, N=3 to assess the angular uniformity in case of an isolated S particle between three L particles and N=4 in case of an isolated S particle between four L particles and of the middle point of an S particle dimer between four L particles).

The goal of the third investigation consists in determining how S particles are arranged relative to L particles. First, the S nearest neighbors to an L particle are determined by Voronoi tessellation/Delaunay triangulation. Subsequently, the number of L particles surrounded by S particles (i.e., having at least one S nearest neighbor) and the number of L particles “caged” by S particles are computed. A particle L is considered to be caged by S particle nearest neighbors if a triangle constructed starting from any three S nearest neighbors out of an arbitrary number of nearest neighbors of the L particle meets the following conditions:

- The center of the L particle lies within the triangle;
- All sides of the triangle (i.e., the center-to-center distances of the three S nearest neighbors) are smaller than $d_{S}+d_{L}$.

In addition, we examined the angular and radial uniformity of the S particles around the L particles.

For the investigation of the angular uniformity, we computed a bond orientational order parameter $\psi_{N_{\mathrm{nnjSL}}}^{\left(j\right)}$ defined as follows:

(2) $$\psi_{N_{\mathrm{nnjSL}}}^{\left(j\right)}=\frac{1}{N_{\mathrm{nnjSL}}}\sum_{k=1}^{N_{\mathrm{nnjSL}}}e^{N_{\mathrm{nnjSL}}i\phi_{\mathrm{jk}}},$$

where $\phi_{\mathrm{jk}}$ is the angle of the line between the center of the L particle j and the $k^{\mathrm{th}}$ of its $N_{\mathrm{nnjSL}}$ S nearest neighbors with respect to an arbitrary fixed reference axis.

For the assessment of the radial uniformity, the following parameter was estimated:

(3) $$c_{{\mathrm{jN}}_{\mathrm{nnjSL}}}=\frac{1}{N_{\mathrm{nnjSL}}}\sum_{k=1}^{N_{\mathrm{nnjSL}}}\frac{\left|t_{\mathrm{jk}}-{\bar{t}}_{j}\right|}{{\bar{t}}_{j}},$$

where $t_{\mathrm{jk}}$ is the distance between the center of the L particle j and the $k^{\mathrm{th}}$ of its $N_{\mathrm{nnjSL}}$ S nearest neighbors and ${\bar{t}}_{j}$ is the average distance of the $N_{\mathrm{nnjSL}}$ S nearest neighbors from the center of the L particle j.

Image analysis was implemented in Matlab and applied to sets of SEM images acquired for each $N_{S}/N_{L}$ value. Approximately $2\cdot 10^{5}$ L particles were examined for each particle number ratio, resulting in a total of about $2\cdot 10^{6}$ L and S particles for all the data sets.

## 3. Results and Discussion

### 3.1. Quantitative Analysis of S Particle Configurations

**Figure 1:**
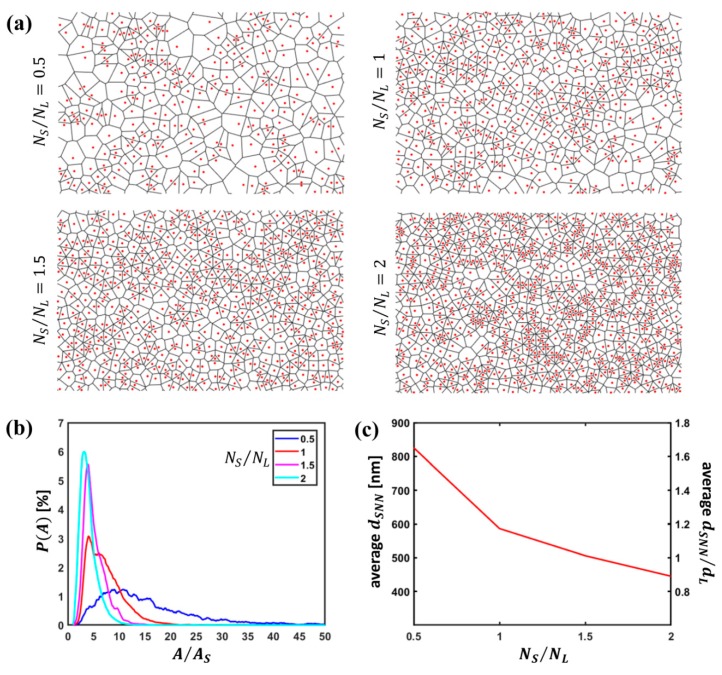
Small particle arrangements and Voronoi diagrams: (**a**) Centroids of S particles and corresponding Voronoi tessellation for variable particle number ratio $N_{S}/N_{L}$; (**b**) probability distribution of the area of the Voronoi cells P(A) normalized to the area of a Voronoi cell of an ideal hcp arrangement of S particles; (**c**) average S nearest neighbor inter-particle distance $d_{\mathrm{SNN}}$.

The red spots in Figure 1a illustrate an example of the determination of the position of the centroids of S particles computed from SEM images for different particle number ratios. As visible, S particles tend to form more densely packed arrangements as $N_{S}/N_{L}$ increases.

As illustrated in the same figure, Voronoi diagrams were constructed from the coordinates of the centroids of the sole S particles. In previous studies [12,49,50,51,52], properties of the Voronoi cells have been investigated to extract information on granular systems and colloidal particle arrangements: for instance, shape factors have been studied within the framework of binary colloids to highlight the transition of L particle arrangements from a predominance of hexagonal order to the coexistence of hexagonal, square and rhombic patterns [12]. Differently from this case, we observe that the sparse character of the distribution of S particles results in Voronoi cells with a broad shape distribution, which does not allow one to infer useful information about particle configurations in contrast to what occurs for L particles that still retain a dense, even if more disordered, distribution. A preliminary quantitative insight into the arrangement of S particles can be gained by computing the probability distribution of the area of the Voronoi cells constructed from the sole S particle centroids P(A) and the average inter-particle distance between S particle neighbors $d_{\mathrm{SNN}}$ for variable $N_{S}/N_{L}$ (Figure 1b,c); the area of the Voronoi cells was normalized to $A_{S}=\frac{\sqrt{3}}{2}d_{S}^{2}$, i.e., the area of a Voronoi cell corresponding to a periodic hexagonal close packing (hcp) of S particles. As visible, an increase in the S particle concentration corresponds to a denser arrangement of S particles with the formation of tiny groups of closely packed S particles. This feature is revealed by the shape of P(A) that becomes progressively narrower and shifts towards lower A values and by the gradually decreasing $d_{\mathrm{SNN}}$ values. Nonetheless, due to the sparse character of such groups, the area of the Voronoi cells is still far from the value corresponding to a hexagonal close packing.

In order to have a more precise characterization of the morphological properties of S particles, it is necessary to shift from a global collective observation of all the S particles (as occurs for the Voronoi tessellation) to an individual analysis of the properties of the different distinct S particle groups that have emerged in the previous visual inspection.

For this purpose, it is essential to find a tool able to individuate the individual tiny groups as separate individual entities. Intuitively, two groups of S particles can be considered as separate and discrete if the minimum distance R between the centroids of the closest particles of the two groups is above a certain threshold value. It should be noted that, in a limit case, a group of S particles is constituted by a single isolated S particle. For the analysis of our assemblies, we selected as the threshold value for the minimum distance between separate entities $R=\frac{d_{L}}{\sqrt{3}}$, i.e., the distance between two adjacent interstices between closely packed L particles in a hexagonal arrangement. This choice would permit to distinguish as separate entities individual particles located in the interstices between L particles for two periodic arrangements of L particles theoretically achievable for $N_{S}/N_{L}=1$ and $N_{S}/N_{L}=2$ in case of a particle size ratio lower than the one used in our experiments. More specifically, we can individuate single S particles in the interstices between L particles in close square packing for $N_{S}/N_{L}=1$ (in this case, the distance between the S particle centroids would be $d_{S-S}=d_{L}>R$) and single S particles in the interstices between L particles in hexagonal close packing for $N_{S}/N_{L}=2$ (with a distance between S particles of $d_{S-S}=R$).

As described in Section 2, on the grounds of this concept of minimum threshold distance, individual entities are identified based on the cardinality of the set of neighbors defined for each S particle j and on the identification of connected components. The set of neighbors contains all the S particles that are within the threshold distance R from the S particle j. Connected components can be identified by constructing a binary image from the coordinates of S particle centroids by drawing discs of radius equal to half of the selected threshold distance centered on such coordinates. Isolated particles, dimers and chains are first identified with the sole cardinality of the set of neighbors. For the identification of clusters, a binary image is built using S particle coordinates and discarding those already recognized as belonging to the other groups; discs of radius R/2 centered in such coordinates are drawn. In this way, each of the individual entities constitutes a connected component; the particles whose centroids belong to the same connected component are regarded as part of the same entity. A final check based on the concept of neighborhood is carried out into the clusters thus identified; clusters made up of particles all having only two nearest neighbors are classified as loops.

**Figure 2:**
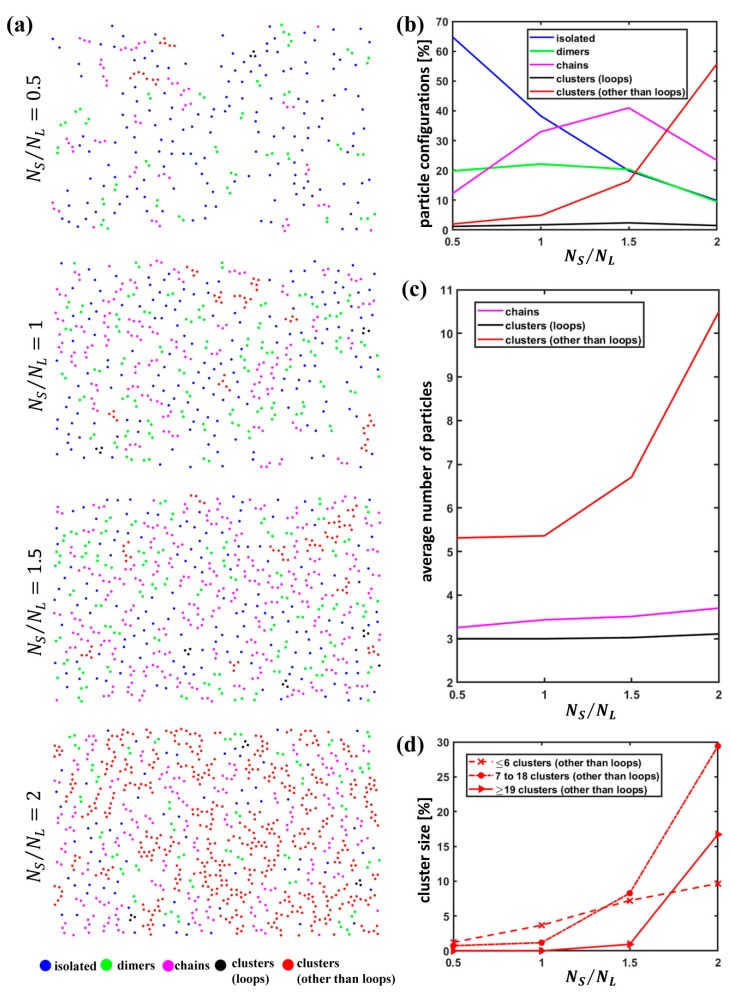
Classification of small particle arrangements: (**a**) Classification and color-coded representation of isolated particles, dimers, chains and clusters for variable particle number ratio $N_{S}/N_{L}$; (**b**) percentage of the different S particle configurations for variable particle number ratio $N_{S}/N_{L}$; (**c**) average number of S particles involved in chains, clusters identified as loops and clusters other than loops for variable particle number ratio $N_{S}/N_{L}$; (**d**) cluster distribution according to the size for variable particle number ratio $N_{S}/N_{L}$.

Figure 2a reports groups of particles color-coded according to whether they were categorized as isolated particles, dimers, chains, clusters classified as loops and clusters other than the loops identified from the examples of SEM images acquired at variable particle number ratio. For an increasing S particle concentration, the number of isolated S particles decreases, while the number of S particles involved in other groups comprising more than one particle appears to rise.

**Figure 3:**
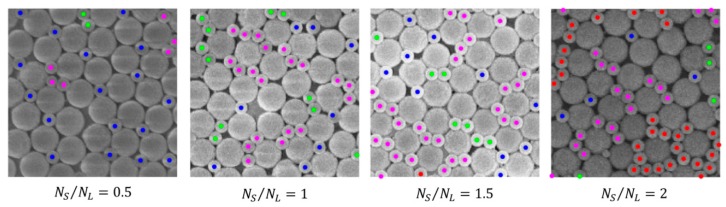
Close-up of binary colloidal assemblies obtained at the air/water interface for variable particle number ratio $N_{S}/N_{L}$; a colour-coded representation of S particles according to the defined categories of classification is superposed.

Figure 3 shows a close-up of SEM images on which a color-coded representation of S particles according to the previous classification is superposed.

As visible, very diverse entities were found in our assemblies, even with strongly anisotropic characteristics, such as chains and clusters. The latter do not typically exhibit a compact isotropic arrangement, even at higher $N_{S}/N_{L}$ values, as, at a visual inspection, they usually present a denser area of few particles surrounded by tails with an anisotropic arrangement.

Our procedure allows the identification of clusters in more disordered and sparse assemblies, which is not affordable with methods used for the individuation of more crystalline clusters (with nearly hexagonal or square symmetry). Such techniques are based, for instance, on setting specific thresholds on diverse combinations of different parameters, such as the magnitude of the bond orientational order parameter, of the bond length deviation (i.e., the variation in the distance between neighboring particles) and of the variation of bond orientation between neighboring particles [6,8,53,54]. However, the groups of S particles identified in our assemblies are far from exhibiting a compact and uniform distribution and, hence, traditional tools for cluster identification would not be adequate in this case. Even a recently developed technique used to distinguish between low-density (gas) and high-density phases without specific assumptions on their symmetry [41] is not applicable for the sparse and anisotropic assemblies encountered in our experiments: in fact, it relies on the regularity of the Voronoi cells and implies a more isotropic distribution of particles (in contrast to the highly anisotropic characteristics of the entities occurring in our assemblies) and clusters of larger size in comparison to those detected in these experiments.

For a quantitative analysis of S particle arrangements for variable $N_{S}/N_{L}$, we evaluated the percentage of S particles involved in the different types of groups identified as isolated particles, dimers, chains, loops and clusters other than loops over the total number of S particles (Figure 2b). It is evident that the number of S particles classified as isolated plummets for an increasing S particle concentration; the percentage of particles involved in loops is low at all particle number ratios, while clusters other than loops climb until becoming dominant at $N_{S}/N_{L}=2$.

Subsequently, the average number of particles composing chains, loops and clusters other than loops was investigated (Figure 2c). As apparent, a rise in the S particle concentration does not entail a significant upturn in the size of loops, which means that loops observed in our assemblies are formed by only few particles independently of the particle number ratio. Also the size of chains exhibits a weak upsurge for increasing $N_{S}/N_{L}$. On the contrary, clusters other than loops display a sharp rise and tend to include a growing number of S particles with a marked boost especially at $N_{S}/N_{L}=2$.

For a deeper insight into the characteristics of groups identified as clusters other than loops, we determined the percentage of S particles involved in different size classes. In this case, the thresholds for the definition of the different size classes were selected as 7 (corresponding to the size of a cluster formed by a particle surrounded by a single shell of nearest neighbors in a hexagonal arrangement) and 19 (corresponding to the size of a cluster made up of one particle and two shells of nearest neighbors in a hexagonal arrangement). The latter value was deemed as the minimum size of a grain in other works [55]. As visible in Figure 2d, even if the cluster size tends to increase at higher S particle concentrations, the clusters of size N≥19 are non-negligible only for $N_{S}/N_{L}=2$ and, even at this value, the dominant size class is between 7 and 18. As observed, at such moderately low S particle concentrations, L particles still preserve a dense packing, preventing the formation of large S particle clusters.

We further scrutinized clusters of size N≥19 at $N_{S}/N_{L}=2$ in order to determine whether they form groups with a uniformly dense packing and gain insight into their structure. For this purpose, we used an analysis based on Minkowski functionals.

Minkowski functionals or measures are a set of topological and geometrical descriptors useful for the characterization of spatial patterns as they provide morphological measures for size, shape and connectivity in D dimensions. Minkowski functionals are computed for patterns constructed starting from the coordinates of the particle centroids by placing a cover disc with radius $R_{m}$ at each couple of coordinates similarly to what we have previously shown in the procedure for the identification of clusters [56,57]. In two dimensions, as in our case, there are three functionals that can be computed from such patterns, i.e., the surface area A of the connected components, the perimeter c and the Euler characteristic $\chi_{E}=N_{C}-N_{H}$, i.e., the difference between the number of connected surfaces $N_{C}$ and the number of holes $N_{H}$ [56,57,58]. The Minkowski functionals are evaluated by varying $R_{m}$, giving rise to a curve characteristic of a given particle configuration [56,57,58]. The dependence on $R_{m}$ of such measures can cast light on the actual morphology of a colloidal assembly. As such, they have been used, for instance, in the analysis of crystalline, hexatic and liquid phases in single-sized colloidal assemblies [57], or in the investigation of binary assemblies of superparamagnetic particles by computing them for the sole L particles and for the sole S particles [56]. Particularly interesting for the purpose of morphological analysis is the Euler characteristic normalized to the number of particles. This Minkowski functional is equal to 1 for small $R_{m}$ (when discs do not touch and no hole is present), can become negative upon a further increase in $R_{m}$ (when discs start to overlap and holes are formed) and starts to rise again for larger $R_{m}$ until approaching 0 for $R_{m}\to \infty$ (when the plane is entirely covered by overlapping discs and holes collapse); specific features for each morphology reverberate into specific values for onsets of fall and rise, characteristic kinks or plateaus [56]. For example, in a perfect hcp pattern of single-sized particles of diameter d, the normalized Euler characteristic is 1 until $R_{m}$ is below d/2 and tends to 0 when $R_{m}$ reaches $d/\sqrt{3}$ [57].

**Figure 4:**
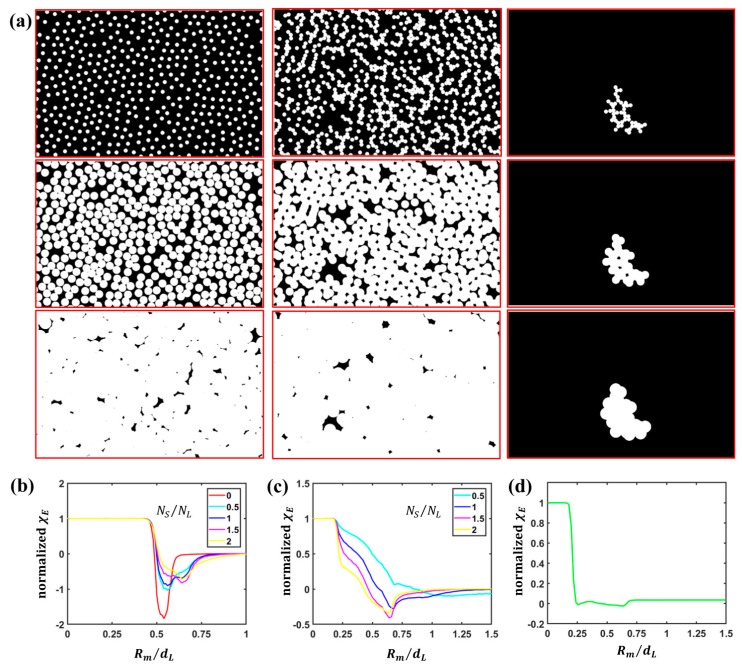
Minkowski functional analysis of small particle arrangements: (**a**) Graphical representation of the construction of patterns for Minkowski functional analysis for an increasing cover disc radius $R_{m}$ in case of L particles (left column), S particles (central column) and S particle clusters of size N≥19 (right column); (**b**) normalized Euler characteristic $\chi_{E}$ computed for L particles for variable particle number ratio $N_{S}/N_{L}$; (**c**) normalized Euler characteristic $\chi_{E}$ computed for S particles for variable particle number ratio $N_{S}/N_{L}$; (**d**) normalized Euler characteristic $\chi_{E}$ computed for S particle clusters of size N≥19.

Figure 4a shows the procedure for the construction of binary images for increasing $R_{m}$ used for the evaluation of the Euler characteristic computed separately on all the L particles (left column), on all the S particles (central column) and on an individual S particle cluster of size N≥19 (right column).

First, we computed the Euler characteristic for the whole set of L particles (Figure 4b) and for the whole set of S particles (Figure 4c) for increasing particle number ratio $N_{S}/N_{L}$. For L particles, the Euler characteristic computed in the case of hcp monolayers obtained for self-assembly of single-sized colloids composed of sole L particles ($N_{S}/N_{L}=0$) was added for reference. The trends resemble those observed for binary assemblies of superparamagnetic particles reported in previous studies [56], and confirm the gradual loss of the hexagonal order of L particles in favor of the coexistence of different symmetries. Interestingly, the Euler characteristic for S particles at higher S particle concentrations shows a peculiar dependence with the presence of a first dip, close to $d_{S}/2$ (i.e., $0.22d_{L}$), when holes between more closely packed particles are formed, followed by a slight increase corresponding to the closure of such holes as the radius of the discs increases, and then a second dip corresponding to the formation of new holes at higher $R_{m}$. Such a trend has been ascribed to the anisotropic character of S particle groupings [56]. However, the analysis of all the S particles hides the features of individual S particle clusters and does not allow one to discern if such a trend is related to the shape of the individual clusters as separate independent entities or only to the formation of holes due to overlap of discs built around particles belonging to different entities.

In order to gain insight into the shape of individual clusters, we applied the analysis to the sole clusters composed of S particles of size N≥19 for $N_{S}/N_{L}=2$. As explained, our goal consists in the description of the morphology of such clusters as individual entities. In this sense, we need to focus individually on each cluster. As a matter of fact, a global analysis of all S particles could bury the specific features of the individual clusters under the collective characteristics of the whole S particle patterns (because connected components and holes would result from the overlap of all the entities previously identified, i.e., isolated particles, chains, loops, other clusters) and obscure the morphology of individual clusters. Figure 4d reports the Euler characteristic computed only for such clusters.

As visible, two regions of fall and rise are present, one at smaller $R_{m}$ values and one at larger $R_{m}$ values. The Euler characteristic exhibits a steep decrease when $R_{m}$ approaches $d_{S}/2$ and subsequently rises. This first region of fall and rise can be attributed to the presence of nuclei of closely-packed S particles and to the opening and closure of holes between such more closely packed S particles. We observe that, for perfect closely packed hexagonal and square patterns, a decrease should occur at $d_{S}/2$ (i.e., $0.22d_{L}$) and an increase at $d_{S}/\sqrt{3}$ and $d_{S}/\sqrt{2}$, respectively (i.e., approximately at $0.25d_{L}$ and $0.31d_{L}$). Afterwards, a second region of fall and rise can be noticed close to $d_{L}/2$. If the particles were more uniformly packed, such a two-step rise in the Euler characteristic would not be observed. For instance, if they formed an hcp pattern, the behavior would be the one previously described. Even in case of less regular and non-closely packed clusters, but still with a more uniformly distributed arrangement, the transition would be more gradual and broader, but it would not exhibit such sharply distinct regions. If we visually inspect a cluster, for instance the one reported in the right column of Figure 4a, we notice how it presents sections composed of particles with a denser packing and sections of particles that appear arranged along the arc of a circle. Euler characteristic analysis confirmed that larger clusters indeed result from the combination of more densely packed arrangements forming in the holes between L particles (that become increasingly large at higher $N_{S}/N_{L}$ values) and by tails that tend to align along the circumference of L particles. Hence, as $R_{m}$ increases, holes begin to form between the discs, resulting in the fall of the Euler characteristic close to $R_{m}=d_{S}/2$. Then, the Euler characteristic starts to rise due to the closure of holes between closer particles in the more densely packed sections of the clusters. New holes form and disappear close to $R_{m}=d_{L}/2$ due to particles aligned along the circumference of L particles.

By the analysis of the Euler characteristic of individual larger clusters, we can therefore obtain an accurate description of the features of their shape that appear buried in the study of the Euler characteristic of the whole ensemble of S particles.

### 3.2. Quantitative Analysis of L Particle Configurations Relative to S Particles

Among the entities previously individuated, two types, i.e., isolated particles and particle dimers, are particularly interesting because, in combination with specific arrangements of neighboring L particles, they fulfil the requirements for potential periodic filling of space.

On the one hand, isolated particles can result in three different forms of periodic structures:

- Isolated S particles present alternatively in the interstices between L particles arranged in a hexagonal pattern;
- Isolated S particles present in the interstices between L particles arranged in a square pattern;
- Isolated S particles present in all the interstices between L particles arranged in a hexagonal pattern.

**Figure 5:**
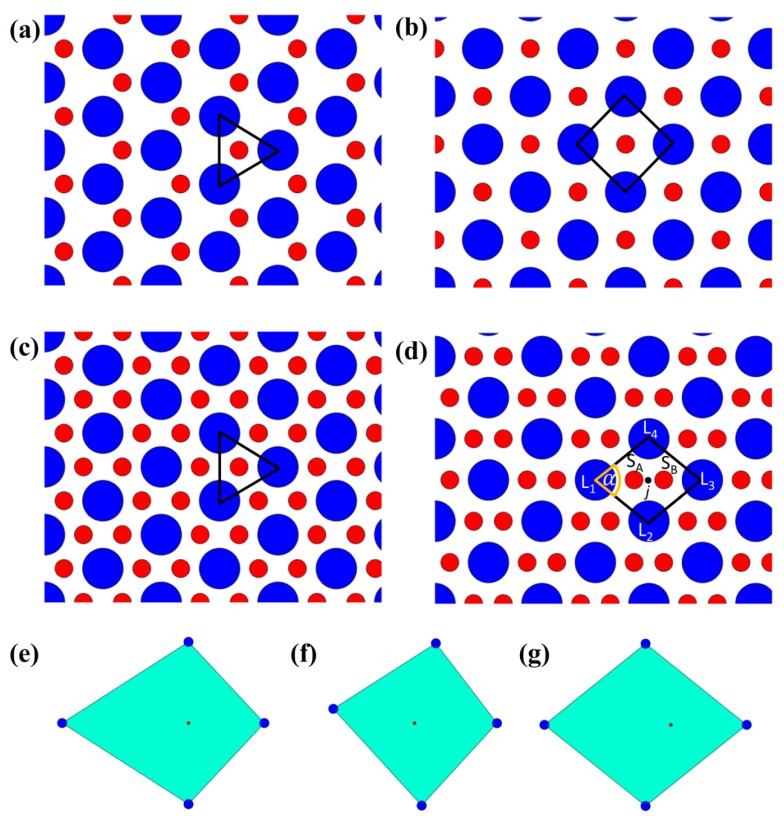
Configurations of L particles around S particles: (**a**) Isolated S particles present alternatively in the interstices between L particles arranged in a hexagonal pattern; (**b**) isolated S particles present in the interstices between L particles arranged in a square pattern; (**c**) isolated S particles present in all the interstices between L particles arranged in a hexagonal pattern; (**d**) S particle dimers present between L particles arranged in a rhombic pattern; (**e**) S particles surrounded by L particles with angularly uniform and radially non-uniform distribution; (**f**) S particles surrounded by L particles with angularly non-uniform and radially non-uniform distribution; (**g**) S particles surrounded by L particles with angularly non-uniform and radially non-uniform distribution.

On the other hand, S particle dimers present in the interstices of L particles located at the vertices of a rhombus can result in a rhombic periodic pattern. Figure 5a–d show a sketch of the different configurations.

Such periodic configurations are theoretically possible for specific values of $N_{S}/N_{L}$ (in particular, the first and the second for $N_{S}/N_{L}=1$ and the third and the fourth for $N_{S}/N_{L}=2$) and for certain ranges of $d_{S}/d_{L}$. The selected value of $d_{S}/d_{L}$ does not allow the formation of periodic hexagonal and square arrangements of L particles; nonetheless, a single occurrence or even repetitions of such cells over tiny areas are frequently encountered even in the amorphous assemblies obtained in our experiments.

In previous studies concerning binary assemblies of superparamagnetic particles, one of such configurations with the potential of periodic space filling, i.e., an isolated S particle between four L particles located at the vertices of a square, was individuated, by studying two different metrics, i.e., the bond orientational order parameter $\psi_{4\mathrm{LS}}^{\left(j\right)}$ and the bond length deviation $b_{\mathrm{jNLS}}$ defined as:

(4) $$b_{\mathrm{jNLS}}=\frac{1}{N}\sum_{k=1}^{N}\frac{\left|l_{\mathrm{jk}}-{\bar{l}}_{j}\right|}{{\bar{l}}_{j}},$$

where $l_{\mathrm{jk}}$ is the distance between the S particle j and the neighbor L particle k and ${\bar{l}}_{j}$ is the average distance between the S particle j and the N L nearest neighbors and N=4 for the square arrangement [8,56,59]. For a perfect square pattern, $\left|\psi_{4\mathrm{LS}}^{\left(j\right)}\right|=1$ and $b_{j4\mathrm{LS}}=0$; to account for a non-ideal arrangement, one S particle and four L particles are considered part of a square arrangement if $\left|\psi_{4\mathrm{LS}}^{\left(j\right)}\right|$ exceeds a certain threshold value and if $b_{j4\mathrm{LS}}$ is below another threshold value.

In our case, we developed a generalized procedure to identify all the different configurations. Such a procedure is based on the analysis of the angular and radial uniformity of L particles around an isolated S particle or around the middle point of an S particle dimer. First, L nearest neighbors are determined via Voronoi tessellation/Delaunay triangulation. Subsequently, for isolated S particles and the middle points of S particle dimers located within the convex polygons having as vertices three or four L particle neighbors, the angular and radial distribution of L particle neighbors with respect to the isolated S particle or to the middle point of the S particle dimer are scrutinized. The goal of the investigation is to check whether they meet the specific conditions satisfied by the ideal arrangements we are searching for. Such analyses aim at assessing whether the isolated S particle or the middle point of the S particle dimer is located approximately at the incenter of such polygons.

In particular, for a single S particle placed within the triangle formed by three L neighbor particles, we computed $\psi_{3\mathrm{LS}}^{\left(j\right)}$ and $b_{j3\mathrm{LS}}=\frac{1}{3}\sum_{k=1}^{3}\frac{\left|l_{\mathrm{jk}}-{\bar{l}}_{j}\right|}{{\bar{l}}_{j}}$ to investigate the angular and radial uniformity, respectively. For the incenter of a perfect equilateral triangle, one should get $\left|\psi_{3\mathrm{LS}}^{\left(j\right)}\right|=1$ and $b_{j3\mathrm{LS}}=0$.

For a single S particle and for an S particle dimer placed within the convex quadrilateral generated by four L neighbor particles, we computed $\psi_{4\mathrm{LS}}^{\left(j\right)}$ for the study of the angular uniformity. As to the radial uniformity, we observe that, for the general case of a rhombus (the square is a degenerate form of rhombus), the four L particles do not exhibit a radially uniform distribution around the incenter (except for the degenerate case of a square), but rather they are pairwise equidistant from the incenter; hence, we defined the following quantity:

(5) $$b_{j4\mathrm{LSdiag}}=\frac{1}{4}\left[\sum_{k=1,3}\frac{\left|l_{\mathrm{jk}}-{\bar{l}}_{1,3}\right|}{{\bar{l}}_{1,3}}+\sum_{k=2,4}\frac{\left|l_{\mathrm{jk}}-{\bar{l}}_{2,4}\right|}{{\bar{l}}_{2,4}}\right],$$

where the four neighbor L particles are ordered in a counter-clockwise direction around the S particle j (in case of isolated S particles) or around the middle point j between two S particles (in case of particle dimers); $l_{\mathrm{jk}}$ is the distance between the S particle j or the middle point j between two S particles and the nearest neighbor L particle k, ${\bar{l}}_{1,3}$ and ${\bar{l}}_{2,4}$ are the average bond lengths relative to the S particle j or to the middle point j between two S particles evaluated at the two couples of L nearest neighbors in alternated positions (see Figure 5d). Such a definition allows one to consider not only squares, but also rhombi, which have diagonals of different lengths. For the incenter of a perfect rhombus, one should get $\left|\psi_{4\mathrm{LS}}^{\left(j\right)}\right|=1$ and $b_{j4\mathrm{LSdiag}}=0.$ Figure 5e shows the case of a point (small red dot) placed at the intersection of the diagonals of a kite having as vertices the four big blue dots: in this case, the condition on angular uniformity $\left|\psi_{4\mathrm{LS}}^{\left(j\right)}\right|=1$ is satisfied, while the one on radial uniformity $b_{j4\mathrm{LSdiag}}=0$ is not satisfied. For the case reported in Figure 5f, the reverse situation is observed, as such an arrangement complies with the conditions on radial uniformity, but it does not abide by the condition on angular uniformity.

As observed, for the detection of such configurations, the position of the isolated S particle or of the middle point of the S particle dimer with respect to the L particles is crucial. This means that an equilateral triangle or a rhombic arrangement of L particles is not sufficient, if such particles do not simultaneously exhibit proper angular and radial distribution around the isolated S particle or the middle point of the S particle dimer. This is the case, for instance, of Figure 5g, where the four big blue dots are arranged in a rhombus, but the small red dot is not located in the incenter of such a rhombus: neither the condition on angular uniformity nor the one on radial uniformity are satisfied. For the detection of specific arrangements of L particles independently of the position of S particles, a persistent homology approach can be used [12]. Here, we focus on a different problem, i.e., the detection of specific arrangements of L particles with respect to S particles and the coordinates of the centroids of both L and S particles play a role.

In addition to the individuation of rhombic arrangements, we proceeded to the classification of their angle in order to understand whether they exhibit a mainly rhombic or square-like arrangement depending on the angle α reported in Figure 5d. To this end, the ratio between the diagonals of such rhombic arrangements (degenerating in squares in the limit case) can be evaluated. Alternatively, the shape factors $\vartheta_{\mathrm{jLS}}$ of the rhombic arrangements having as vertices four L particles and including the isolated S particle j or the middle point j of an S particle dimer can be estimated for the determination of α. The shape factor $\vartheta_{\mathrm{jLS}}$ relative to an isolated S particle or to the middle point of an S particle dimer j can be written as:

(6) $$\vartheta_{\mathrm{jLS}}=\frac{p_{\mathrm{jLS}}^{2}}{4{\pi A}_{\mathrm{jLS}}},$$

where $p_{\mathrm{jLS}}$ and $A_{\mathrm{jLS}}$ represent the perimeter and the area of the rhombus having at the vertices the four L nearest neighbors to an isolated S particle or to the middle point of an S particle dimer j.

These metrics allow us to infer the angle α of the rhombi and to distinguish them into three different classes, respectively with $60^{{}^{\circ}}\leq \alpha \leq 67.5^{{}^{\circ}}$, $67.5^{{}^{\circ}}<\alpha <82.5^{{}^{\circ}}$ and $82.5^{{}^{\circ}}\leq \alpha \leq 90^{{}^{\circ}}$. The first class corresponds to rhombi degenerating in a configuration of L particles corresponding to a nearly hexagonal arrangement; in the case of closely packed L particles, the interstice between the four L particles would degenerate into two separate interstices for $\alpha =60^{{}^{\circ}}$; an isolated S particle, for such a configuration of L particles, would be located along the bridge between two neighbor L particles, i.e., along the line connecting the centers of two L particles in contact. The second class would correspond to a more rhombic-like arrangement, while the third class to a more square-like arrangement.

The combination of all these analyses allows a full characterization of the configurations of L particles relative to isolated S particles and S particle dimers.

**Figure 6:**
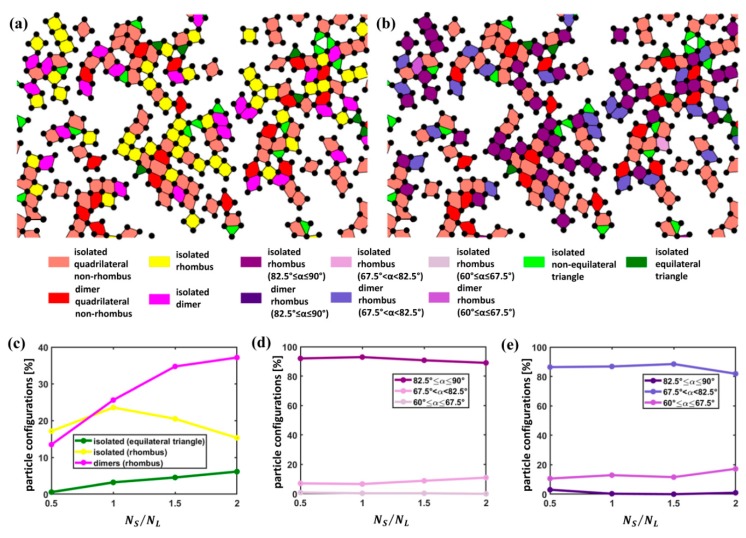
Angular and radial uniformity of quadrilaterals and triangles of L particles with respect to isolated S particles and S particle dimers: (**a**) Identification of isolated S particles involved in equilateral triangles and rhombi and of S particle dimers involved in rhombi; (**b**) classification of rhombi around S particles and S particle dimers according to the angle α; (**c**) percentage of isolated S particles involved in equilateral triangles and rhombi and of S particle dimers involved in rhombi for variable particle number ratio $N_{S}/N_{L}$; (**d**) angle classes of rhombi around isolated S particles for variable particle number ratio $N_{S}/N_{L}$; (**e**) angle classes of rhombi around S particle dimers for variable particle number ratio $N_{S}/N_{L}$.

Figure 6a reports a color-coded representation of the analysis of triangles and convex quadrilaterals of L neighbors surrounding isolated S particles and convex quadrilaterals of L neighbors surrounding S particle dimers. Configurations individuated as equilateral triangles and rhombi are highlighted in specific colors. To account for the non-ideal arrangements of particles, the requirements on angular and radial uniformity were loosened: triangles were assigned an equilateral triangle-like character if $\left|\psi_{3\mathrm{LS}}^{\left(j\right)}\right|\geq 0.9$ and $b_{j3\mathrm{LS}}\leq 0.05$; similarly, convex quadrilaterals were classified as rhombic-like if $\left|\psi_{4\mathrm{LS}}^{\left(j\right)}\right|\geq 0.9$ and $b_{j4\mathrm{LSdiag}}\leq 0.05$. In Figure 6b, rhombi are color-coded according to the angle α.

For a quantitative comparison of the dominant configurations at variable particle number ratio $N_{S}/N_{L}$, the percentage of isolated S particles located approximately at the incenter between L particles placed at the vertices of an equilateral triangle or of a rhombus was computed with respect to the total number of isolated S particles. Similarly, the percentage of S particle dimers whose middle point is roughly at the incenter of a rhombus of L particles was determined with respect to the total number of S particle dimers for different $N_{S}/N_{L}$ values. The results of the aforementioned analysis are reported in Figure 6c. As visible, for isolated S particles, rhombic-like arrangements exhibit a peak at $N_{S}/N_{L}=1$ and equilateral triangles steadily rise for increasing $N_{S}/N_{L}$; for S particle dimers, the percentage of rhombi shows a remarkable increase up to nearly 40% for $N_{S}/N_{L}=2$.

For the analysis of the angle α of the rhombic arrangements, we considered the percentage of rhombi belonging to the different classes of angle α with respect to the total amount of rhombi for isolated S particles (Figure 6d) and S particle dimers (Figure 6e) for variable $N_{S}/N_{L}$. Concerning the isolated S particles, for all the S particle concentrations, the dominant configuration is a square-like one ($82.5^{{}^{\circ}}\leq \alpha \leq 90^{{}^{\circ}}$), followed by a rhombic-like configuration ($67.5^{{}^{\circ}}<\alpha <82.5^{{}^{\circ}}$); the presence of configurations with $60^{{}^{\circ}}\leq \alpha \leq 67.5^{{}^{\circ}}$ is only fractional. As to particle dimers, the prevailing pattern corresponds to a rhombic-like arrangement ($67.5^{{}^{\circ}}<\alpha <82.5^{{}^{\circ}}$); in this case, particle dimers involved in a square-like arrangement with $82.5^{{}^{\circ}}\leq \alpha \leq 90^{{}^{\circ}}$ are insignificant.

Overall, our results demonstrate the dominance of square-like arrangements for $N_{S}/N_{L}=1$ for isolated S particles and of rhombic-like arrangements for $N_{S}/N_{L}=2$ for S particle dimers. 

In conclusion, the approach described in this paragraph allows a comprehensive characterization of the configurations of L particles relative to S particles and the detection of configurations of interest by the analysis of the angular and radial distribution of L particles around S particles and of metrics for the determination of the shape of rhombic-like arrangements to distinguish between more square-like and more rhombic-like ones.

### 3.3. Quantitative Analysis of S Particle Configurations Relative to L Particles

The goal of this investigation consists in determining how S particles are arranged with respect to L particles and to understand how such arrangements evolve with a variation in S particle concentration.

**Figure 7:**
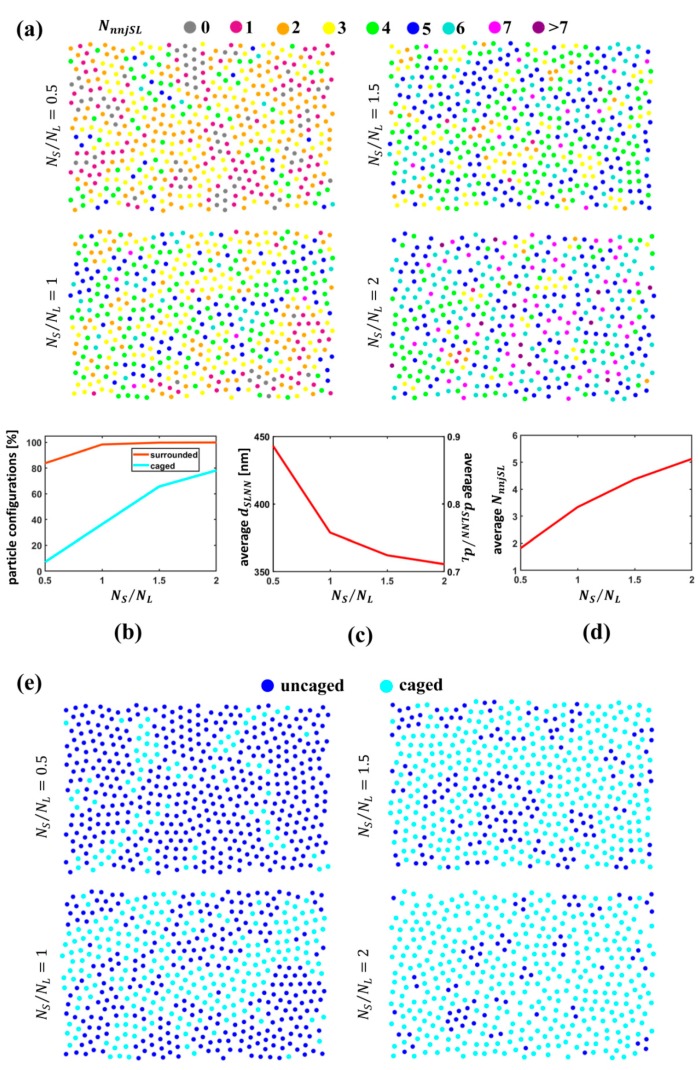
Characterization of S particles with respect to L particles: (**a**) Color-coded representation of L particles according to the number of S nearest neighbors $N_{\mathrm{nnjSL}}$ for variable particle number ratio $N_{S}/N_{L}$; (**b**) percentage of L particles surrounded by at least one S particle ($N_{\mathrm{nnjSL}}\geq 1$) and of caged L particles for variable particle number ratio $N_{S}/N_{L}$; (**c**) average distance of S nearest neighbors to an L particle from the L particle $d_{\mathrm{SLNN}}$; (**d**) average number of S nearest neighbors $N_{\mathrm{nnjSL}}$ for variable particle number ratio $N_{S}/N_{L}$; (**e**) color-coded representation of caged and uncaged L particles for variable particle number ratio $N_{S}/N_{L}$.

As a first step, we determined the S particle nearest neighbors $N_{\mathrm{nnjSL}}$ surrounding an L particle for variable $N_{S}/N_{L}$. S particle nearest neighbors were determined via Voronoi tessellation/Delaunay triangulation constructed from the coordinates of both S and L particles. Figure 7a shows a color-coded representation of L particles according to the number of S nearest neighbors for increasing $N_{S}/N_{L}$. Figure 7b–d report the percentage of L particles having at least one S nearest neighbor (line in brown), the average distance of the centroids of the S particle neighbors from the L particles $d_{\mathrm{SLNN}}$ and the average number of nearest neighbors to each L particle. We observe that, for all S particle concentrations, most of L particles have at least one S nearest neighbor; however, S particles become closer to L particles and the average number of S particles surrounding L particles climbs dramatically upon an increase in the S particle number from half to twice the number of L particles. This means that S particles tend to surround L particles for increasing $N_{S}/N_{L}$ rather than forming separate large clusters, as could occur, for instance, in the limit case of segregation of L and S particles [2].

To gain more accurate insight into the specific arrangements of such particles and in particular to assess the uniformity of the distribution around L particles, we adopted two approaches.

**Figure 8:**
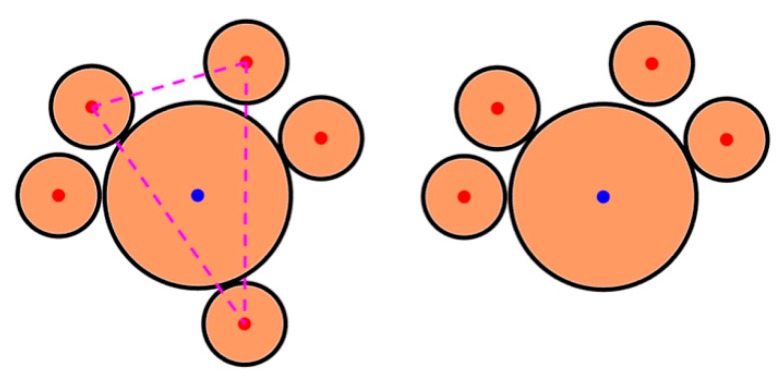
Graphical representation of the concept of caged and uncaged particles: the large particle illustrated on the left is caged by the three small particles forming the triangle with dotted magenta sides; the large particle on the right is not caged by any combination of small particles.

To give a complementary perspective on the distribution of S particles around L particles, we computed the percentage of L particles “caged” by S particles. The concept of caging in colloidal science has been introduced to study percolation in amorphous assemblies of single-sized colloidal particles [52,60]. Here, we resorted to this concept to individuate those configurations that are likely not to make an L particle “escape” from the set of S nearest neighbors. Figure 7e shows a color-coded representation of L particles caged by S particles; the percentage of caged particles surges dramatically from approximately 10% to nearly 80% upon an increase in the S particle number from half to twice the number of L particles (cyan line in Figure 7b). To clarify the concept of caging, a sketch of a caged and of an uncaged particle is reported in Figure 8. As visible, the S particles that constitute a cage for the L particle exhibit a more uniform spatial distribution around the L particle, while those that do not cage the L particle are concentrated in just one area and are not homogeneously arranged around the L particle. Hence, the concept of caging allows one to identify only configurations that correspond to a more uniform distribution of S particles enclosing an L particle; the computation of the percentage of caged L particles and of its variation with the particle number ratio provides immediate insight into the spatial uniformity of S particles around L particles.

**Figure 9:**
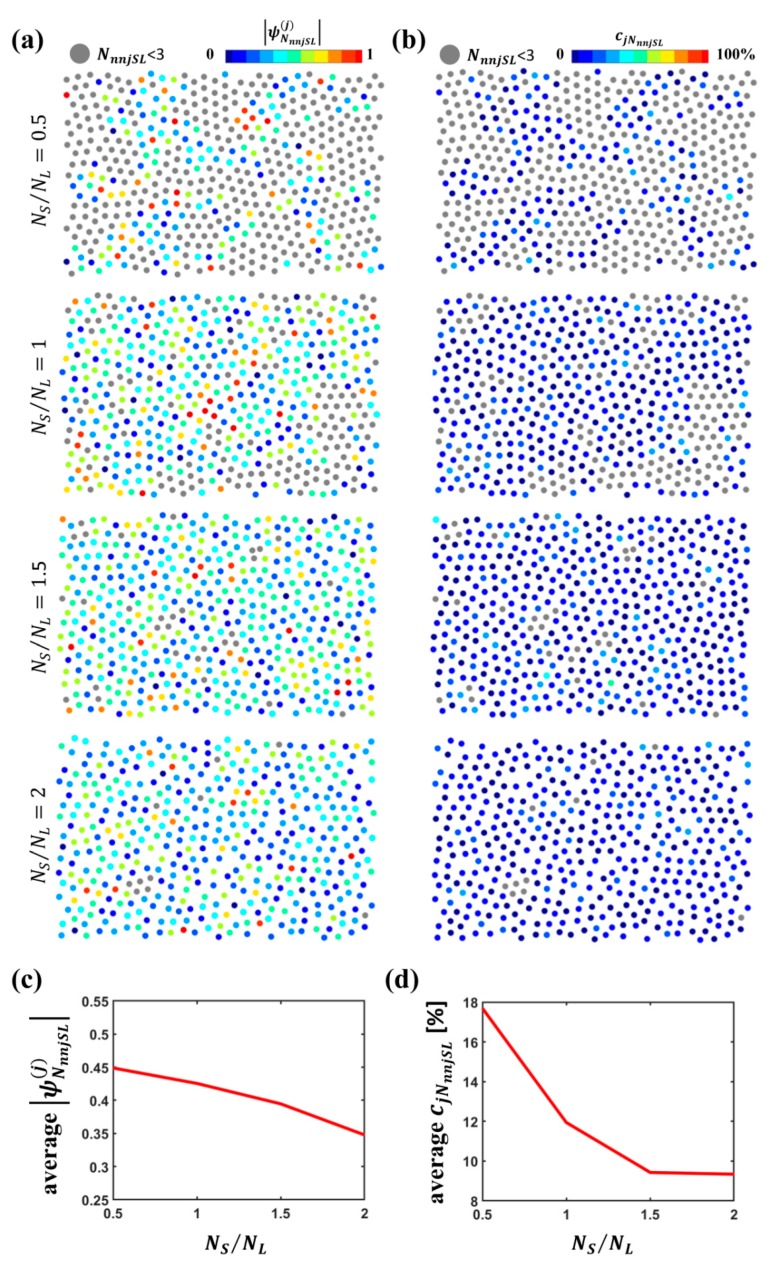
Angular and radial uniformity of S particles with respect to L particles: (**a**) Identification of angular uniformity and color-coded representation of $\left|\psi_{N_{\mathrm{nnjSL}}}^{\left(j\right)}\right|$ for variable particle number ratio $N_{S}/N_{L}$ for L particles surrounded by at least three S nearest neighbors; (**b**) identification of radial uniformity and color-coded representation of $c_{{\mathrm{jN}}_{\mathrm{nnjSL}}}$ for variable particle number ratio $N_{S}/N_{L}$ for L particles surrounded by at least three S nearest neighbors; (**c**) average value of $\left|\psi_{N_{\mathrm{nnjSL}}}^{\left(j\right)}\right|$ for variable particle number ratio $N_{S}/N_{L}$; (**d**) average value of $c_{{\mathrm{jN}}_{\mathrm{nnjSL}}}$ for variable particle number ratio $N_{S}/N_{L}$.

Finally, for all L particles having a number $N_{\mathrm{nnjSL}}$ of S particle nearest neighbors equal at least to 3, we assessed the angular and radial uniformity by computing the bond orientational order parameter $\psi_{N_{\mathrm{nnjSL}}}^{\left(j\right)}$ and the radial uniformity parameter $c_{{\mathrm{jN}}_{\mathrm{nnjSL}}}$. Figure 9a,b provide a picture of L particles color-coded according to the value of $\left|\psi_{N_{\mathrm{nnjSL}}}^{\left(j\right)}\right|$ and $c_{{\mathrm{jN}}_{\mathrm{nnjSL}}}$, respectively; L particles indicated in gray are those with $N_{\mathrm{nnjSL}}<3$, for which $\psi_{N_{\mathrm{nnjSL}}}^{\left(j\right)}$ and $c_{{\mathrm{jN}}_{\mathrm{nnjSL}}}$ were not computed. A value of $\left|\psi_{N_{\mathrm{nnjSL}}}^{\left(j\right)}\right|$ closer to 1 denotes a more regular angular distribution of S particles around the L particle, whereas a value of $c_{{\mathrm{jN}}_{\mathrm{nnjSL}}}$ closer to 0 indicates a more uniform radial distribution. Figure 9c,d report the value of $\left|\psi_{N_{\mathrm{nnjSL}}}^{\left(j\right)}\right|$ and $c_{{\mathrm{jN}}_{\mathrm{nnjSL}}}$ averaged over all the L particles with at least three S nearest neighbors.

As visible, for an increasing S particle concentration, the radial uniformity of the distribution of S particle nearest neighbors around the L particles is enhanced at higher S particle concentrations, while the angular uniformity is rather low at all $N_{S}/N_{L}$ values and exhibits a slight drop for rising S particle concentrations. These results are compatible with the scenarios outlined in the investigation of the arrangements of the sole S particles with the presence of entities of different types, such as isolated particles, dimers, chains, loops and strongly anisotropic S particle clusters, exhibiting nuclei of more closely packed particles and tails that create arcs surrounding L particles. The set of S nearest neighbors to an L particle is composed of S particles belonging to different entities. At higher $N_{S}/N_{L}$ values, entities including a larger number of particles increase and tails of clusters tend to form arcs around L particles, implying a denser packing around L particles, which justifies a better radial uniformity of their distribution around L particles. However, this does not entail a better angular uniformity. In fact, such arrangements of S particles do not necessarily result from large loops surrounding L particles, but may, for instance, derive from arcs of particles aligned along the rim of L particles belonging to different entities and, hence, with a non-uniform angular distribution. For example, one L particle may be surrounded by an arc of close S particles belonging to a chain and to a separate arc constituting the tail of a cluster; while all such particles will be mostly equidistant from the center of the L particle resulting in a good radial uniformity, their angular distribution will be non-uniform.

In brief, for increasing $N_{S}/N_{L}$, S particles tend to encircle and cage L particles; the distribution of S particle nearest neighbors around the L particles becomes more radially uniform, while angular uniformity is poor in all the cases.

## 4. Conclusions

In this article, we unveiled the evolution of the arrangements assumed by small particles within binary colloidal assemblies, consisting of particles of two different sizes assembled at the air/water interface, as the concentration of small particles increases. To this end, we presented an innovative morphological analysis approach, allowing a comprehensive and detailed quantitative characterization suitable to investigate not only the specific system considered in this study, but also, in general, to gain insight into amorphous and sparse colloidal assemblies of different types.

Sparse colloidal assemblies with non-uniform spatial distribution are characterized by the organization of particles into sub-assemblies or entities with different configurations, which can range from isolated particles to groups of two or several closer particles.

For the system investigated in this work, the tendency of S particles to form such sub-assemblies in the globally sparser arrangement of all S particles is evident from SEM images, but is obscured by analyses based, for instance, on the properties of the Voronoi cells, which do not provide proper insight into the morphological properties of the individual particle groups. While previous analyses have provided some indications about the characteristics of such clusters of S particles in binary assemblies [12,56], a comprehensive and systematic characterization requires a shift of the focus from the global analysis of the whole ensemble of S particles to the study of the characteristics of the individual sub-assemblies, which imposes the need for a methodical and precise identification of such entities.

The effective method proposed in the article is based on a precise definition of diverse classes of particle configurations or groupings (i.e., isolated particles, dimers, chains, clusters defined as loops and clusters other than loops) and is particularly suitable for particle arrangements of a sparse and non-spatially uniform nature. Once a minimum distance to identify such sub-assemblies as individual entities is defined, the procedure is based on the concepts of connected components and neighborhood: all the different separate entities that were identified as possible configurations assumed by the particles (i.e., isolated particles, dimers, chains, loops and clusters other than loops) were classified according to their peculiar and specific features with respect to these concepts. All the individual entities are unambiguously identified according to a univocal definition based on the concepts of connected components once a minimum distance between particles belonging to distinct entities is defined; the concept of neighborhood was used to classify such entities into one of the particle configuration classes. For instance, loops are connected components characterized by the fact that all the particles have two nearest neighbors. Each particle in the sparse assembly can hence belong to only one entity and can be clearly assigned to each of these individual entities with a specific configuration class and no particle can belong simultaneously to more than one sub-assembly. This effective approach allowed the identification, characterization and quantification of the diverse forms of particle sub-assemblies or entities in a clear and univocal manner.

In this way, we can attain a systematic characterization of sparse and disordered colloidal assemblies, which fail to be accurately characterized by other analysis tools suitable for ordered assemblies with a dominant specific symmetry. Traditional cluster identification tools [6,8,53,54] are inadequate in this case as they imply specific assumptions on the symmetry (e.g., square or hexagonal) of the clusters, not applicable to S particle assemblies which are sparse and disordered. Nor are suitable procedures based on the analysis of the regularity of Voronoi cells [41] associated to S particles, due to the highly sparse and anisotropic character of the S particle sub-assemblies often composed of few particles. Even more sophisticated tools based, for example, on persistent homology [12], developed for amorphous and disordered systems, do not allow a full characterization of such sparse disordered systems, despite being appropriate for denser particle arrangements.

Once the different sub-assemblies are identified, the collective characteristics of the whole sparse colloidal arrangement in specific experimental conditions can be studied in terms of the relative weight of the different entities or sub-assemblies (i.e., the percentage of particles involved in the different classes), and analyzed with further morphological characterization tools. Such additional characterization of the individual entities involves the determination of the size of the different entities (i.e., of the number of particles involved in chains, loops and clusters other than loops). In addition, the morphology of specific entities can be scrutinized using Minkowski functionals, in particular the computation of the Euler characteristic, to cast light on their shape for specific entities.

By applying this innovative procedure which enables the recognition and quantification of different types of particle configurations, we studied the evolution of the arrangements of small particles in amorphous binary colloidal assemblies upon variation in the experimental conditions, e.g., the concentration of S particles. In particular, we revealed the transition from a regime where isolated particles are dominant at low small particle concentrations to the prevalence of sub-assemblies, like chains and clusters of increasing size, at higher small particle concentrations. The scrutiny of the configurations assumed by the sole S particles demonstrated that a growth of $N_{S}/N_{L}$ from 0.5 to 2 is accompanied by a progressive increase in the weight of dimers, chains, loops and clusters relative to isolated particles that are predominant at low $N_{S}/N_{L}$; in addition, the average size of clusters increases significantly. Nonetheless, the investigation of the shape of clusters involving at least 19 particles (corresponding to the minimum size of a cluster formed by a particle surrounded by two shells of nearest neighbors in a hexagonal arrangement) using the Euler characteristic revealed that even such larger clusters do not take on a compact, dense and isotropic structure, but are rather formed by a denser core of a few particles placed in the holes between L particles and tails that tend to surround L particles by aligning along their rims in arcs of circumference. In fact, at moderately low particle number ratios, the distribution of L particles is still relatively dense, preventing the formation of very large and compact S particle clusters. Hence, in spite of the increasing size of such S clusters, their structure is not compact and uniform, but rather anisotropic. The Euler characteristic computed for larger clusters uncovered features that could be masked by the application of the same analysis to the whole ensemble of particles and elucidated their highly anisotropic shape.

To get a complete description of small particles within the binary assemblies, we analyzed the relative configurations assumed by one type of particle relative to the other (e.g., L particles relative to S particles and vice versa). For this purpose, we developed specific metrics aimed at assessing the angular and radial uniformity of one particle species with respect to the other. In this way, it was possible to gain deeper insight into the characteristics of small particle sub-assemblies and to individuate the experimental conditions for the prevalence of specific mutual arrangements of interest.

In particular, the analysis of the configurations assumed by L particles with respect to S particles aimed at individuating specific arrangements with the potential of periodic space filling for $N_{S}/N_{L}=1$ and $N_{S}/N_{L}=2$, namely isolated S particles in the interstices between three L particles arranged in an equilateral triangle, isolated S particles in the interstices between four L particles arranged in a square, S particle dimers in the interstices between four L particles arranged in a rhombus. Even if such configurations were not found on a large scale in our binary assemblies due to the fact that we selected a value of $d_{S}/d_{L}$ resulting in an amorphous pattern, an isolated occurrence or small repetitions of such cells were encountered. The analysis revealed a conspicuous presence of square arrangements around isolated S particles for $N_{S}/N_{L}=1$ and of rhombic arrangements around S particle dimers for $N_{S}/N_{L}=2$.

Finally, the investigation of the arrangements of S particles relative to L particles confirmed the fact that S particles tend to encircle and cage L particles for increasing $N_{S}/N_{L}$. Even if S particles tend to become closer and more equidistant to the L particles for higher S particle concentrations, their angular distribution around L particles remains non-uniform.

In brief, in this article, we presented a detailed quantitative characterization of small particles in amorphous binary colloidal assemblies, a system which is drawing increasing attention in the field of colloid science for a broad range of applications, for instance, in optics and acoustics [11,17,19,23].

In addition, we proposed a morphological analysis approach, which is of great interest for a precise morphological characterization of a wide range of colloidal assemblies. The implications of the current work are not limited exclusively to the analysis of binary assemblies because the techniques developed for the identification and characterization of particle sub-assemblies are remarkable for an ample range of particle assemblies. Sparse assemblies characterized by the presence of chains, rings and small clusters have been encountered in very different contexts within the field of colloid science [31,33,34,35,37]. Such arrangements have been demonstrated to influence, for instance, the optical properties of colloidal assemblies [36]. Hence, as such more complex colloidal assemblies are of interest for the design of novel colloidal materials, it is of primary importance to have tools for their analysis and characterization. The powerful approach presented in this article has a potential tremendous impact on the analysis and design of colloidal materials with accurately tailored physical properties and is of great interest for the investigation of a wide range of particle assemblies.

Beyond the design of novel colloidal materials, our method can be used to identify structural features of colloidal systems and correlate them with physicochemical processes occurring during particle assembly in specific experimental conditions. In this sense, our quantitative morphological characterization approach can provide insight into colloidal interactions and mechanisms of particle assembly and help to investigate the fundamental properties of colloidal assemblies.

In addition, our approach could be extended to the investigation of three-dimensional (3D) structures, so far mostly investigated with a combination of real-space local structure analysis (based on microscopy images with a resolution at the single particle level) relying on traditional techniques (such as the bond orientational order parameter) and reciprocal space techniques (based on scattering measurements, which typically average over many particles) [61,62,63,64].

Furthermore, we remark that the techniques developed to identify the configurations of S particles relative to L particles and of L particles relative to S particles have enabled an extensive classification of a broad spectrum of configurations of interest in the field of binary colloids, with the potential extension of the analysis of the study of radial and angular uniformity to the investigation of composite colloidal assemblies with different symmetry.

The approach elaborated in the study can therefore be applied to the analysis of an extensive gamut of colloidal assemblies investigated not only with SEM, but also via other techniques, for instance, atomic force microscopy and scanning near field optical microscopy [65,66,67,68,69,70]. Applications to nanoscale and atomic systems could be envisaged due to developments in high resolution transmission electron microscopy and other techniques, which can provide real-space local structure imaging besides the more widespread reciprocal space information inferred from scattering experiments [71,72].

In conclusion, we provided a comprehensive characterization of the evolution of structural transitions in S particle arrangements in binary colloidal assemblies and developed a method of general interest for the morphological characterization of sparse colloidal assemblies, which may mark a significant headway in the search for robust methods for the identification, classification, description and quantification of colloidal patterns.
